# The glymphatic system: a new perspective on brain diseases

**DOI:** 10.3389/fnagi.2023.1179988

**Published:** 2023-06-15

**Authors:** Zhao Ding, Xiaodi Fan, Yehao Zhang, Mingjiang Yao, Guangrui Wang, Yilei Dong, Jianxun Liu, Wenting Song

**Affiliations:** Beijing Key Laboratory of Pharmacology of Chinese Materia Medic, Institute of Basic Medical Sciences of Xiyuan Hospital, China Academy of Chinese Medical Sciences, Beijing, China

**Keywords:** the glymphatic system, cerebrospinal fluid, perivascular spaces, astrocytes, AQP-4

## Abstract

The glymphatic system is a brain-wide perivascular pathway driven by aquaporin-4 on the endfeet of astrocytes, which can deliver nutrients and active substances to the brain parenchyma through periarterial cerebrospinal fluid (CSF) influx pathway and remove metabolic wastes through perivenous clearance routes. This paper summarizes the composition, overall fluid flow, solute transport, related diseases, affecting factors, and preclinical research methods of the glymphatic system. In doing so, we aim to provide direction and reference for more relevant researchers in the future.

## 1. Introduction

It is traditionally believed that the lymphatic system doesn’t exist in the central nervous system (Nedergaard and Goldman, 2016). Thus, cell debris, potential neurotoxic proteins, and other metabolites with large molecular weight are considered to be removed in a different clearance pathway than brain vasculature. In 2012, the Nedergaard (Iliff et al., 2012) group found that cerebrospinal fluid (CSF) can enter brain parenchyma and exchange with brain interstitial fluid (ISF) in the presence of aquaporin-4 (AQP4) on astrocytes. Likewise, when the mixed fluid was drained from the brain, Amyloid-β (Aβ) was transported along with the outflow. Since the function of this “drainage” pathway is similar to that of the lymphatic system and supported by astrocytes, it was named the glymphatic system sooner after glia (Jessen et al., 2015).

Since last decade, many researchers in the field of neurology, neurodegenerative diseases and physiology have aimed to study and develop the glymphatic system, providing a brand-new perspective for us to understand brain diseases: the overall fluid flow of the brain rather than a specific lesion or structure. Herein, we summarized the components of the glymphatic system, the fluid circulation mode within this system, how pathogenic solutes are transported in certain diseases, affecting factors of its function, and by what means we can study the glymphatic system. All above may provide direction and reference for brain diseases and medical researchers.

## 2. The glymphatic system in physiological conditions

### 2.1. The composition of the glymphatic system

The glymphatic system is a brain-wide perivascular pathway driven by AQP4 on astrocytic endfeet, which can deliver nutrients and neuroactive substances to the brain parenchyma through peri-arterial CSF influx pathway and remove metabolic wastes through peri-venous clearance routes (Gouveia-Freitas and Bastos-Leite, 2021). This system is mainly composed of three elements: peri-arterial CSF influx realized by AQP4 on astrocytes, infusion of CSF and ISF in the brain parenchyma, and peri-venous clearance routes (Szczygielski et al., 2021). Therefore, the function of the glymphatic system is closely related to two structures: the Perivascular Space (PVS) and AQP4 on astrocytes.

#### 2.1.1. The perivascular space

**FIGURE 1:**
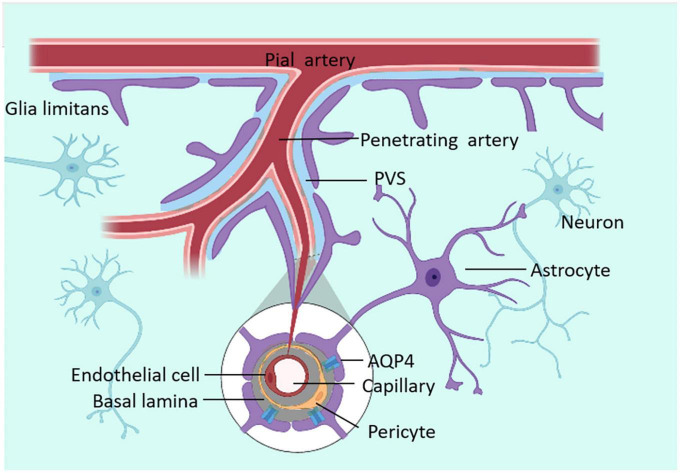
The structure of VRS. VRS is an annular tunnel space around penetrating arterioles in the brain parenchyma, filled with CSF. CSF can enter the brain parenchyma through PVS.

In the 19th century, Rudolf Virchow and Charles Robin found annular tunnel spaces around penetrating arterioles in the brain parenchyma and named them Virchow-Robin spaces (VRS). Subsequently, researchers found that all arterioles, capillaries, and venules in the brain parenchyma were surrounded by this structure resembling a donut-shaped tunnel, and called it Perivascular spaces (PVS) (Zhang et al., 1990). The inner wall of PVS comprises vascular cells (mostly endothelial cells and smooth muscle cells), while the outer wall is built by perivascular astrocytic endfeet (Yao D. et al., 2022). VRS is connected with space around pial arteries, and the liquid it is composed of is CSF (Jessen et al., 2015). As the penetrating arterioles narrow deeper down in the brain parenchyma, the CSF-containing PVS becomes continuous with the basal lamina. The basal lamina mainly comprises laminin, fibronectin, type IV collagen, and heparin sulfate proteoglycan. Due to the loose structure of the extracellular matrix of these cells, the basal lamina has the least resistance to CSF influx. CSF will flow downwards from the VRS along the peri-arterial space, enter the basal layer surrounding capillaries, and exit along the perivenous space (Jessen et al., 2015). The structure of PVS is shown in Figure 1.

In 2012, researchers injected fluorescent tracers with different molecular weights into the cisterna magna of anesthetized mice, followed by *in vivo* two-photon imaging and immunofluorescence techniques to track the CSF circulation through the brain interstitial space. Results from this study demonstrated that CSF entered brain parenchyma along the PVS and rapidly exchanged with ISF. Then the mixture of CSF and ISF was cleared along the perivenous drainage pathways (Iliff et al., 2012). Notably, this process was supported by AQP4 in astrocytes.

#### 2.1.2. Astrocytes and AQP4

Astrocytes provide a link between blood vessels and neurons. They can transmit neural activities from synapses to the basal lamina, where vascular endothelial cells, pericytes, and astrocytes are located (Lu and Gao, 2022). The increase of Ca^2+^ in astrocytes caused by neurotransmitters leads to the synthesis and release of vasoactive metabolites such as TGF-β, glial-derived neurotrophic factor (GDNF), b-fibroblast growth factor (bFGF), interleukin-6 (IL-6) and angiopoietins, prostaglandin E2 (PGE2), epoxyeicosatrienoic acids (EETs), and 20-hydroxyeicosatetraenoic acid (20-HETE) (Bicker et al., 2014; MacVicar and Newman, 2015), leading to vasoconstriction and vasorelaxation (Burn et al., 2021), further causing changes in PVS space. In addition, there are 20 nm clefts between the endfeet of astrocytes around PVS, allowing macromolecular solutes to pass through (Czigler et al., 2020).

**FIGURE 2:**
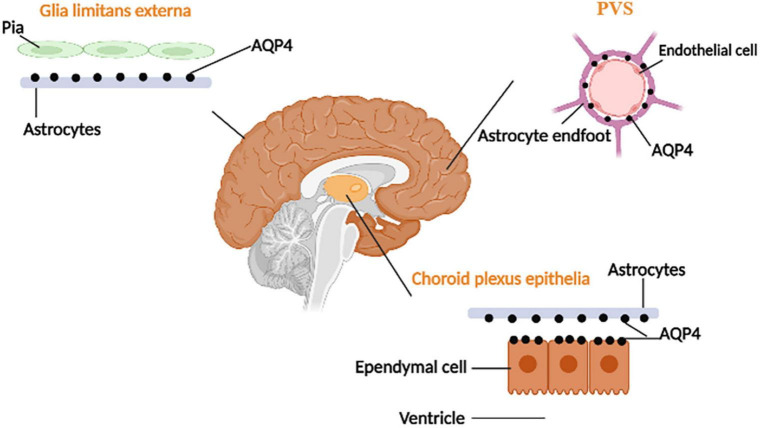
The distribution of AQP-4. AQP4 is located at the ependymal cells and the astrocyte processes (which face CSF–brain and blood–brain barriers).

AQP4s are located in surfaces of the blood–brain barrier and CSF–brain barrier and are expressed in astrocyte endfeet processes surrounding capillaries. Astrocyte processes comprise the subpial and subependymal glial-limiting membranes and the perivascular astrocytic end-foot processes that circumscribe the entirety of the cerebrovasculature (Zhao Z. et al., 2022). The distribution of AQP4 is shown in Figure 2. As one of the essential components of astrocyte endfeet that constitutes the outer wall of PVS, AQP4 mediates fluid and small molecular substances (molecular weight:0∼18; diameter:0∼0.38 nm) in CSF to enter the brain parenchyma (Peng et al., 2023). By injecting several CSF tracers with different molecular weights and contrast medium into AQP4 knock-out (KO) mice or mice that lack perivascular AQP4 (Snta1 KO), there is a significant decrease in CSF tracer transport in KO mice and rats compared to controls. Notably, these findings suggest that AQP4 on the astrocytes supports perivascular CSF influx and ISF outflow in the glymphatic system (Mestre et al., 2018).

**FIGURE 3:**
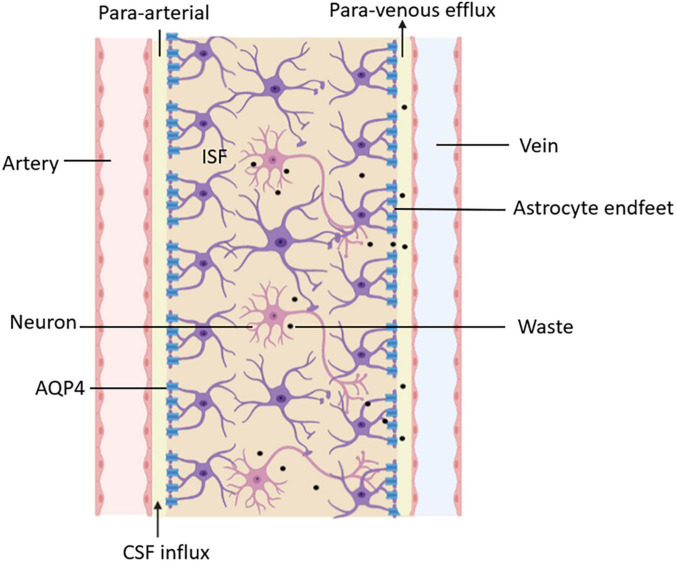
Functional diagram of the glymphatic system. CSF delivers nutrients and neuroactive substances to the brain parenchyma through the peri-arterial spaces pathway and mixes with ISF in the brain parenchyma. The mix of CSF and ISF removes metabolic wastes through peri-venous clearance routes.

Moreover, the glymphatic system is not only a specific tissue or structure but also a functional generalization of the flow and transportation of fluid in the brain. After CSF enters the brain parenchyma, it mixes with ISF, exchanges substances, and clears the metabolites of brain tissue through the perivenular outflow channel. A functional diagram of the glymphatic system is shown in Figure 3. The proposal of the glymphatic system has updated our understanding of fluid circulation in the brain, which is of significant importance.

### 2.2. The glymphatic system and brain fluid circulation

**FIGURE 4:**
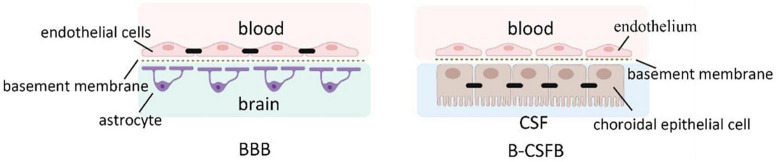
The composition of BBB and B-CSFB. The composition of BBB: A layer of endothelial cells interconnected through tight junctions and not containing fenestrations, basal membrane consisting of the basal lamina of astrocytes and basal lamina of endothelial cells, protrusions of astrocytes (Zhang et al., 2022). The composition of B-CSFB: choroidal epithelial cells interconnected by tight junctions (which are more permeable than the junctions between the endothelial cells of brain capillaries), basal membrane, endothelium of the pia mater capillaries containing fenestrations (Jessen et al., 2015).

**FIGURE 5:**
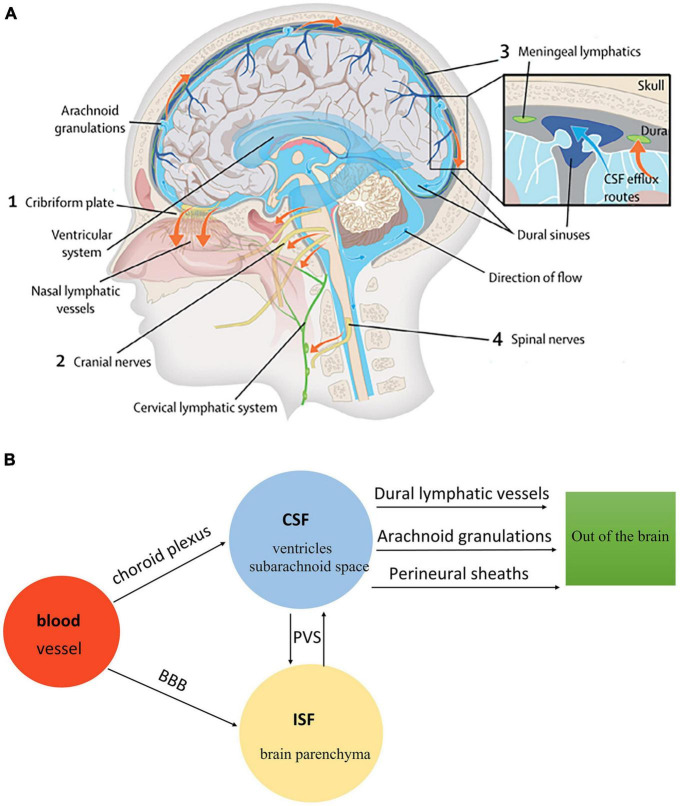
**(A)** The fluid circulation in the glymphatic system (Rasmussen et al., 2018). Blood generates CSF through choroid plexus capillary endothelial cells distributed in the lateral, third, and fourth ventricle. CSF flows from the ventricular system to the subarachnoid space of the brain and spinal cord. CSF in the subarachnoid space enters brain parenchyma through the perivascular spaces of penetrating arteries, which AQP4 drives on the end feet of astrocytes. Additionally, CSF mixes with ISF in brain parenchyma. The mixture of CSF and ISF subsequently enters the perivenous space. Egress sites of cranial fluid (orange arrows) fall into three functional categories: the perineural sheaths surrounding cranial and spinal nerves, dural lymphatic vessels, and arachnoid granulations. Finally, the mixture of CSF and ISF is drained to the cervical lymph nodes. **(B)** The fluid circulation in the glymphatic system.

There are four kinds of fluid in the brain: blood, CSF, ISF, and intracellular fluid. Blood in vessels and ISF in brain parenchyma are separated by the blood-brain barrier (BBB), while blood and CSF are separated by the blood-cerebrospinal fluid barrier (B-CSFB) (Markowicz-Piasecka et al., 2022). The composition of BBB and B-CSFB is shown in Figure 4. Blood generates CSF through choroid plexus capillary endothelial cells distributed in the lateral ventricle, third ventricle, and fourth ventricle and enters the ventricles and subarachnoid space for storage (Saunders et al., 2023). The process of CSF production requires the Na/K-ATPase and aquaporin 1 (AQP1) scattered on the choroid plexus epithelial cells (de Laurentis et al., 2021). Simultaneously, some solutes and water in the blood can enter the brain tissue through the BBB and become parts of ISF. With pial arteries in the CSF-containing subarachnoid space becoming penetrating arteries upon diving into the brain parenchyma (Iliff et al., 2013a), CSF enters the brain parenchyma through the perivascular spaces of penetrating arteries, which AQP4 drives on the endfeet of astrocytes (Mogensen et al., 2021). In brain parenchyma, CSF mixes with ISF and exchanges substances. This indicates that CSF becomes an additional source of ISF through the glymphatic system (Nedergaard and Goldman, 2016). Notably, CSF and ISF exit the brain parenchyma through three pathways: (1) Perineural sheaths surrounding the head and face. CSF enters the nasal mucosa along the olfactory nerve’s nerve sheath towards the nasal mucosa’s lymphatic vessels. From here, the CSF is drained to the cervical lymph nodes. Other perineural efflux pathways in rodents are the trigeminal, glossopharyngeal, vagal, and spinal accessory nerves (Lundgaard et al., 2017). (2) Dural lymphatic vessels. Dural lymphatic vessels distribute on the dura mater, the sigmoid sinus, the retro glenoid vein, the middle meningeal artery, and the pterygopalatine artery (Louveau et al., 2015). Dural lymphatic vessels absorb CSF from the adjacent subarachnoid space and ISF from the glymphatic system and transport fluid into deep cervical LNs (dcLNs) via foramina at the base of the skull (Aspelund et al., 2015). (3) Arachnoid granulations. CSF in the subarachnoid space flows from the arachnoid granulations into the sagittal sinus and is directly discharged into the blood (Pollay, 2010). The fluid circulation is shown in Figure 5.

Traditionally, fluid circulation in the glymphatic system is thought to be driven by pressure difference, respiration, and arterial pulsation (Nakada and Kwee, 2019). CSF is constantly generated in the choroid plexus, creating a pressure that causes fluid to flow from the ventricular to the subarachnoid space. Breathing also plays a vital role in the circulation of CSF. Researchers have found that during deep breathing, each inhalation will cause high CSF flow, and breath holding will inhibit it. Notably, this may be related to the reduction of chest pressure during inhalation (Djukic et al., 2022). When the internal carotid artery was ligated unilaterally, the arterial pulsation was reduced by about 50%, and the rate of CSF-ISF exchange was slowed down. When the adrenergic agonist Dobutamine is used throughout the body, the pulsation of the penetrating artery increases by about 60%, and the rate of CSF-ISF exchange in perivascular is accelerated (Iliff et al., 2013b). These findings indicate that arterial pulsation promotes the flow of CSF in the glymphatic system through the perivascular space. In recent years, the discovery of parenchymal border macrophages (PBMs) located in pia mater and rain arterial tree orders has updated fluid flow dynamics in the brain. The subpopulation of PBMs that expresses high levels of CD163 and LYVE1 (scavenger receptor protein) regulates arterial brain movement and changes CSF flow (Drieu et al., 2022). Pharmacological or genetic depletion of PBMs led to obstructed CSF and accumulated extracellular matrix proteins (Drieu et al., 2022).

The glymphatic system complements the problems that cannot be explained in the traditional CSF circulation theory. For example, in traditional theory, CSF is mainly produced by the choroid plexus (Thompson et al., 2022). In this case, removing the choroid plexus should have a certain effect on patients with hydrocephalus. Still, the clinical data found that removing the choroid has no obvious effect on the treatment of hydrocephalus (Oresković and Klarica, 2010). The concept of a glymphatic system gives the hypothesis that ISF enters the subarachnoid space through PVS and becomes one of the CSF sources. In traditional CSF circulation theory, the elimination of metabolites in the brain is mainly completed by diffusion. But the production and transport of metabolites in the brain are rapid, which is difficult to explain by diffusion (Cserr et al., 1981). Bulk flow in the glymphatic system can properly supplement the traditional theory so that people can understand why substances with different molecular weights are injected into the caudate nucleus and can be eliminated from the brain at almost the same speed.

### 2.3. Solutes transport in the glymphatic system

There are two categories of solutes transported in the glymphatic system: (1) Nutrients (such as glucose and lipids) (Lundgaard et al., 2015) and neuroactive substances (such as transthyretin and apolipoprotein E) (Kassem et al., 2006; Xu et al., 2006); (2) Metabolites in the central nervous system, such as amyloid β(Aβ) and α-Synuclein. Studies have also shown that glucose, lipids, growth factors, neurotransmitters, neuroactive substances, and medicine injected into CSF (such as anti-tumor medicine) can be transported to the brain through the influx of CSF (Kassem et al., 2006; Xu et al., 2006; Rangroo Thrane et al., 2013; Lundgaard et al., 2015). Especially in lipid transport, the glymphatic system plays a central role in the brain. Metabolites in the central nervous system, such as Aβ, α-Synuclein, tau protein, and lactic acid, can also be discharged from the brain through the glymphatic system (Rasmussen et al., 2022). Aβ exists in the brain interstitium, and its abnormal precipitation is closely related to neurodegenerative diseases such as Alzheimer’s (Zhao J. et al., 2022). From investigations, the fluorescence-labeled Aβ is transported along the glymphatic system pathway in the central nervous system. When AQP4 is knocked out, the clearance of Aβ in the central nervous system slows down due to fluids congestion because Aβ is mainly removed by bulk flow along the gliovascular clearance system rather than locally across the BBB (Iliff et al., 2012). For solutes that cannot pass through the BBB, the fluxion of CSF in the glymphatic system may be the only medium for them to inflow and outflow the brain parenchyma. Therefore, the study of the glymphatic system is conducive to exploring how medicine molecules enter the brain parenchyma without passing through the BBB and promote the clearance of brain pathogenic factors, which may benefit neurodegenerative disease therapies.

## 3. The glymphatic system and diseases

### 3.1. Alzheimer’s disease

Alzheimer’s disease (AD) is a neurodegenerative disease mainly characterized by memory impairment and decreases in brain volume (Choi and Tanzi, 2023). The pathogenesis of AD is related to the formation of senile plaques by Aβ (Ma et al., 2017), neurofibrillary tangles caused by abnormal accumulation of tau protein (Reeves et al., 2020), and depolarization of AQP4(AQP4 polarization: AQP4 is mainly expressed in astrocytes’ endfeet; AQP4 depolarization: AQP4 expression is transferred from astrocytes’ endfeet to the whole astrocytes cell body) on the astrocytes (Lynch et al., 2022). In APP/PS1 mice (a model of AD), toxic Aβ (such as soluble oligomers) accumulation is related to malfunction of glymphatic system transport, which is quantified by the solute inflow into the brain from CSF and clearance from the brain by radio-labeled tracers. Notably, decreased glymphatic system transport is associated with Aβ40 entering and accumulating in the perivascular spaces, which is related to AQP4 depolarization (Iliff et al., 2012). Thus, a malfunction of the glymphatic system preceded significant amyloid-β deposits, which may be an early signal of AD (Uchida, 2022).

### 3.2. Parkinson’s disease

Parkinson’s disease (PD) is a chronic neurodegenerative disease related to unbalanced production and clearance of α-syn (Scott-Massey et al., 2022). When meningeal lymphatic drainage was blocked by ligating the deep cervical lymph nodes of A53T mice (PD model), the glymphatic inflow of CSF tracer in the mice’s brain was reduced, which resulted in more severe α-syn accumulation, neuroglia activation, inflammation, loss of dopaminergic neurons and dyskinesia (Zou et al., 2019). The deletion of AQP4 inhibited the clearance of α-syn in the brain, manifested by the increase of protein monomers but not the increase of oligomers (Bobela et al., 2015). These indicate that AQP4 and meningeal lymphatic drainage are partially responsible for clearance of α-syn soluble monomers, suggesting a promising direction for PD symptom alleviation.

### 3.3. Stroke

Stroke is mainly divided into hemorrhagic and ischemic stroke, characterized by local nerve dysfunction due to cerebral blood circulation disorder (Xu et al., 2023). Hemorrhagic stroke includes cerebral and subarachnoid hemorrhage, and ischemic stroke includes cerebral infarction and cerebral thrombosis (Li and Chen, 2022). After 24h of subarachnoid hemorrhage, immunohistochemical results showed that fibrin clots blocked the perivascular spaces of cerebral cortical vessels (Pu et al., 2019), and the glymphatic system function could be repaired by intraventricular injection of tissue plasminogen activator (t-PA) (Bosche et al., 2020). This indicates that subarachnoid hemorrhage leads to disorders of the glymphatic system and related pathological damage. Notably, targeted improvement of cerebral glymphatic clearance may become a new strategy for treating hemorrhagic stroke. In the ischemic stroke model, pathological changes similar to subarachnoid hemorrhagic stroke can be observed, such as the expansion of perivascular space, AQP4 polarity distribution changed from normal perivascular pattern to scattered parenchymal pattern (Back et al., 2017; Wang et al., 2017). Therefore, protecting and maintaining the normal function of glymphatic system will help attenuate stroke injury and also help improve cognitive impairment due to stroke.

### 3.4. Brain edema

Brain edema is a pathological phenomenon of brain tissue damage caused by increased brain volume and cranial pressure due to excessive intracellular or intercellular fluid. Brain edema is traditionally divided into two phases: early cytotoxic and later vasogenic phases (Mestre et al., 2020). In cytotoxic edema, energy deficiency caused by hypoxia promotes CSF influx in the glymphatic system and inhibits ISF efflux (Thrane et al., 2014). Additionally, AQP4 protein expression positively correlates with the degree of brain edema (Mamtilahun et al., 2019). Moreso, vasogenic brain edema is mainly caused by the destruction of the BBB and the increase of capillary permeability, resulting in a large amount of liquid entering the extracellular fluid. Importantly, AQP4 is considered the mechanism of water removal from the interstitial spaces, and vasogenic edema and intracranial pressure can be improved by regulating the role of AQP4 (Zhou et al., 2022). The conclusions of AQP4 in the cytotoxic and vasogenic edema models are entirely different, suggesting that AQP4 may be a selective two-way water channel. Moreover, liquid flow direction depends on whether the BBB is intact: when the BBB remains intact, AQP4 transports water into the ISF, and when the BBB is damaged, it becomes reverse transport (Assentoft et al., 2015). However, the mechanism of this process needs to be explored.

### 3.5. Traumatic brain injury

Traumatic brain injury (TBI) is caused by brain tissue trauma, which leads to brain dysfunction (Sivandzade et al., 2020). Following brain injury, astrocytes can release several vasoactive substances, such as isoprostanes (vasoconstrictors of cerebral arterioles) and endothelin 1 (causes vasoconstriction related to calcium influx), which could cause decreased cerebral perfusion (Jullienne et al., 2016). Notably, TBI causes the accumulation of Aβ and tau protein (Hicks et al., 2022), which is a risk factor for neurodegenerative diseases (such as dementia, AD, etc.) (Gao et al., 2022). Likewise, injury in mice’s brain parenchyma causes the loss of polarity distribution of AQP4 on the endfeet of astrocytes. As a result, the function of the glymphatic system decreases by about 60% and lasts for at least one month (Iliff et al., 2014). These indicate that chronic injury of the glymphatic system after TBI may be a key factor leading to tau protein aggregation and neurodegenerative attacks in the brain after trauma. Therefore, further exploration of the glymphatic system can help find ways to alleviate TBI and prevent the transformation to degenerative diseases after TBI.

### 3.6. Multiple sclerosis

Multiple sclerosis (MS) is a chronic immune dysfunction disease characterized by inflammation and demyelinating plaques (Dobson and Giovannoni, 2019). The characteristic pathological marker of MS is perivenous inflammatory lesions, causing demyelinating plaques (Karussis, 2014). Non-invasive diffusion MRI was used to calculate the diffusion along the perivascular space index (a proxy for glymphatic function). Notably, results showed that the diffusion along the PVS in patients with multiple sclerosis was lower, and this lower diffusion index was associated with more severe clinical disabilities and longer disease duration (Carotenuto et al., 2022; Verghese et al., 2022). Research has also found that MS patients are more likely to detect enlarged perivascular spaces in MRI and suggested that using CSF diversion to decongest perivascular spaces, which possibly improves glymphatic function and interstitial removal of proinflammatory molecules from CSF, finally downregulating local inflammation (Granberg et al., 2020; Scollato et al., 2022). The depolarization of AQP4 was found in the cuprizone-induced demyelination model and chronic MS lesions of post-mortem human subjects, which may be related to metabolic injury to the brain (Rohr et al., 2020). These findings suggest that the function of the glymphatic system is impaired during the process of MS, particularly in progressive stages, which implies that the glymphatic system is deeply involved in multiple sclerosis.

### 3.7. Amyotrophic lateral sclerosis

Amyotrophic lateral sclerosis (ALS) is a progressive degenerative motor neuron disease characterized by the selective death of motor neurons (Giovannelli et al., 2022). Although the underlying cause of ALS is not yet fully understood, its pathological mechanisms involve proteostasis, glutamate excitotoxicity, dysregulation of RNA metabolism, nuclear-cytoplasmic transport, and autophagy. More than 97% of ALS cases show the presence of insoluble proteins such as TDP-43 and tau protein in the brain. Furthermore, tau protein elevation in CSF indicates upper motor neuron degeneration, leading to ALS (Süssmuth et al., 2010). Therefore, promoting the clearance of tau protein in the glymphatic system may be an effective way to treat ALS. When astrocytes are exposed to ALS-CSF, their morphology is changed and accompanied by increased GFAP reactivity, further acquiring neuroinflammatory features (Mishra et al., 2016). Sleep, which plays an essential role in the clearance of the glymphatic system, is also disrupted in patients with amyotrophic lateral sclerosis (Boentert, 2019). Thus, these findings suggest that further exploring the functional mechanism of the glymphatic system will be helpful to better explain the physiological and pathological mechanism of ALS.

### 3.8. Cognitive impairment associated with diabetes

Diabetic patients often have cognitive and memory decline, and are at higher risk of vascular diseases and AD (Li Z. et al., 2022). MRI analysis of Type-2 diabetic rats showed the clearance rate of contrast agent Gd-DTPA in CSF from interstitial space in the hippocampus was three times slower than that of non-DM rats (diabetes rat model), which was confirmed by fluorescence imaging analysis (Jiang et al., 2017). These results indicate Type-2 diabetes can inhibit the exchange flow of CSF and ISF, leading to impaired glymphatic system function. The impairment may be due to the expansion of the perivascular space. The metabolic wastes accumulated in expanded perivascular space will trigger an inflammatory reaction, thus causing further expansion of perivascular space (Yao X. Y. et al., 2022). The study of the glymphatic system provides a new explanation for cognitive impairment caused by diabetes, but its mechanism is still unclear and requires further experiments to warrant.

### 3.9. Migraine

A migraine is a recurrent unilateral or bilateral fluctuating headache. Cortical spreading depression (CSD) is a potential signal that waveform can be characterized by an intense neuronal activity that slowly progresses over the cortex and a period of neuronal inactivity (Pi et al., 2022). Acute puncture and KCl were used to induce transient CSD in mice. CSD can induce quick closure of penetrating periarterial and perivenous space on the cortical surface, lasting for several minutes and gradually recovering within 30 min (Schain et al., 2017). During CSD, the common occurrence of white matter lesions in headaches results from liquid and waste retention in the interstitial space in the distended perivascular space (Toriello et al., 2021). Therefore, accelerating the flow of CSF to improve the clearance of wastes in the perivascular space may be a new direction for treating migraine.

The discovery of the glymphatic system gives us a new understanding of the efficient fluid transport and metabolite clearance of the central nervous system, which can help us better understand the pathogenesis of diseases and find new ways to treat them. Notably, the abnormal accumulation of Aβ (Ma et al., 2017), tau protein (Reeves et al., 2020), and depolarization of AQP4 on the astrocytes(the decrease of AQP4 on the endfeet of paravascular astrocytes) (Back et al., 2017; Wang et al., 2017) may be involved in the occurrence and aggravation of the diseases. Thus, further approaches will be explored to address these disease conditions.

## 4. Influencing factors of the glymphatic system

### 4.1. Sleep

The function of the glymphatic system is partly influenced by the sleep-wake cycle. In the sleep state, the function of the glymphatic system is significantly enhanced than in the awake. CSF inflow of mice in sleep and awake states were compared by in vivo two-photon imaging technology, and results showed that, within 30 minutes after CSF tracer injecting, the tracer inflow of the same mice in the awake state was 95% lower than that in the sleep state (Xie et al., 2013). Moreover, the volume fraction of brain interstitial space in sleeping mice (22∼24%) was significantly higher than that in awake mice (13∼15%) (Xie et al., 2013). The inhibition of CSF inflow during wakefulness may be partly related to noradrenaline (NE) secretion. NE is a neuromodulator that regulates the activity of neuronal and non-neuronal cells (Murtazina et al., 2021). Notably, it can reduce the interstitial space by increasing the cell volume, thus increasing the resistance of the inflow of CSF in the interstitial space (Sherpa et al., 2016; Hablitz et al., 2019). In addition, sleep deficiency can also lead to a decrease in the clearance rate of metabolic proteins in the brain parenchyma, resulting in protein deposition such as Aβ and tau protein (Shokri-Kojori et al., 2018). In short, increasing sleep time or improving sleep quality can enhance CSF inflow and metabolite clearance.

### 4.2. Aging

Many neurodegenerative diseases (such as AD, PD, ALS, and so on) often occur in middle and old age, and their pathogenesis is associated with the accumulation of Aβ and tau protein (Jessen et al., 2015). Aβ and tau protein can be cleared from brain parenchyma with CSF outflow in the glymphatic system. The clearance rate of interstitial solutes was compared between young (2–3 months old) and old (18–20 months old) wild-type mice, and results showed that Aβ clearance was reduced by 40%, accompanied by a 27% decrease in the pulsatility of the vascular wall of the arterioles in the old mice cortex, as well as a widespread loss of AQP4 polarization along the penetrating artery (Kress et al., 2014). In addition, aging leads to artery stiffening, which can reduce arterial pulsatility in the cerebral cortex of the elderly, in turn inhibiting CSF inflow from the PVS to the brain parenchyma (Zieman et al., 2005). This indicates that with the growth of age, the functions of the glymphatic system will deteriorate, affecting the clearance of metabolic wastes in the brain.

### 4.3. Anaesthesia

The function of the glymphatic system is closely related to the anesthetics selected in the experiment, and different anesthetics play a role in promoting or inhibiting. In the awake state, CSF tracers injected into mice cisterna magma hardly enters brain parenchyma (Benveniste et al., 2019). However, after intraperitoneal injection of ketamine/xylazine (KX), CSF rapidly rushed into the brain parenchyma along the periarterial space, with speed comparable to that of naturally sleeping mice, and CSF inflow increased significantly (Xie et al., 2013). Moreso, the effects of different anesthetics were compared. The CSF tracer influx was highest under K/X, followed by isoflurane (ISO) supplemented with dexmedetomidine and pentobarbital. The mice anesthetized with α-chloralose, Avertin, or ISO exhibited low CSF tracer influx. Additionally, it was found that under anesthesia, CSF inflow in the glymphatic system correlates positively with cortical delta power (neuronal network oscillations) in electroencephalogram (EEG) recordings and negatively with heart rate (Hablitz et al., 2019). Additionally, the cortical delta power may contribute to the fluid influx into the brain parenchyma and clearance of waste from the brain, and increased in naturally sleeping animals (Benveniste et al., 2017). Therefore, in the design of CSF flow experiments, we should pay attention to the selection and use of anesthetics to avoid side effects.

### 4.4. Body position

Different body positions can also affect the function of the glymphatic system during sleep or anesthesia. The transport efficiency of the glymphatic system in the lateral position of anesthetized rats was higher than that in the supine and prone positions by dynamic-contrast-enhanced MRI and kinetic modeling. The tracer in the brain of prone rats entered and cleared slowly in the glymphatic system (Lee et al., 2015; Muccio et al., 2021). Optical imaging and radiotracer studies also confirmed that the transport of the glymphatic system and the clearance rate of Aβ were faster in the lateral and supine positions (Lee et al., 2015). The changes in body position may affect the function of the cardiovascular and respiratory systems and subsequently impact the pulsation leading to CSF/ISF circulation (Muccio et al., 2021). This mechanism needs to be further explored. These findings suggest that the lateral position during sleep/anesthesia may be advantageous in removing brain metabolic wastes, including Aβ. In the future, we should also pay attention to the placement of the test animals in the experimental design of the glymphatic system.

### 4.5. Alcohol

Alcohol influences the glymphatic system in two different ways. Glymphatic system function was significantly inhibited after mice were exposed to 1.5 g/kg (binge level) ethanol acutely and chronically (Lundgaard et al., 2018). Chronic exposure to 1.5 g/kg ethanol increased GFAP expression (associated with astrocyte reactive phenotype and reduced glymphatic function (Iliff et al., 2014; Kress et al., 2014)) and induced mislocation of the astrocyte-specific AQP4. The function of the glymphatic system increased in mice treated with 0.5 g/kg (low dose) ethanol following acute exposure. After one month of chronic exposure, low doses of chronic ethanol intake were associated with a significant decrease in GFAP expression (Lundgaard et al., 2018). Low-dose ethanol also has a vasodilatory effect, which may promote the interaction of endothelial, smooth muscle cells by activating the production of endothelial-specific nitric oxide synthase (eNOS) and vasodilator nitric oxide, thus promoting the diffusion of metabolic wastes in the central nervous system to the perivascular space (Cheng et al., 2019). Therefore, low-dose intake of alcohol can be potentially beneficial to the function of the glymphatic system. In contrast, long-term excessive intake of alcohol will inhibit the function of the glymphatic system.

### 4.6. Sports

Sports can improve cognitive ability, especially in patients with vascular degeneration and neurodegenerative diseases. The glymphatic system function of voluntary wheel running and sedentary mice were compared. CSF inflow continuously increased in voluntary exercise mice during the awake period, mainly concentrated in the hypothalamus and ventral parts of the cortex. However, it also occurred in the middle cerebral artery territory. Voluntary exercise increases CSF flux in a wide range of brain regions in mice, which may be related to the role of exercise in promoting cognition (von Holstein-Rathlou et al., 2018). Another study demonstrated that voluntary wheel running accelerated the protein clearance of the glymphatic system rather than the protein penetration of the BBB (He et al., 2017). It also improved the expression and polarization of AQP4 in astrocytes, thereby protecting mice from synaptic dysfunction and spatial cognitive decline, indicating a possible mechanism of exercise-induced neuroprotection in the aging brain (He et al., 2017).

In short, these influencing factors affect the fluid inflow and outflow of the glymphatic system mainly by regulating the cardiovascular and respiratory systems. When arterial pulsation slows down, or respiration is inhibited, the flow of CSF is inhibited, affecting the clearance of macromolecular metabolic proteins in the brain. The accumulation of metabolic proteins in the brain parenchyma further affects the size of the brain interstitial space, thus increasing the resistance to CSF inflow. However, the specific mechanisms involved in this process still need further exploration. These influencing factors suggest that attention should be paid to the patient’s daily sleep, drinking, and exercise behaviors to prevent brain-related diseases. When designing experiments related to the glymphatic system, attention should be paid to the choice of anesthesia, the position, and the age of experimental animals.

## 5. Preclinical methods to study the fluid flow of the glymphatic system

The study of the glymphatic system can be divided into *in vivo* and *ex vivo* techniques. Imaging *ex vivo* is usually performed with light sheet fluorescence microscopy of an observed brain or spinal cord sections from the optically cleared brains of mice receiving CSF tracer injections while under anesthesia (Hablitz et al., 2020; Hauglund et al., 2020). This way, high-resolution (up to 200 nm) 3D imaging of tracer distribution within the cells and in perivascular spaces can be observed (Lohela et al., 2022). Brain or spinal cord fixed coronal sections are usually combined with immunohistochemistry to compare CSF flow with the expression pattern of related proteins. However, in recent years, Panoptic imaging of transparent mice has also been used to analyze the distribution of tracers in the brain (Bèchet et al., 2020). Imaging *in vivo* is mainly utilized to observe the flow of CSF in the brain by injecting a CSF tracer into the cisterna magna and using two-photon fluorescence imaging (TPI) (Iliff et al., 2013b; Tithof et al., 2019), near-infrared fluorescence (NIRF) (Myllylä et al., 2018), transcranial optical imaging (Plog et al., 2018), magnetic resonance imaging(MRI) (Bai et al., 2022; Li C. et al., 2022) and so on. TPI has high spatial resolution and can observe 240μm below the cortical surface, which is conducive to observing the diffusion of the CSF tracer along the outside of cortical surface arteries and penetrating arterioles (Iliff et al., 2012). However, TPI is invasive to the study object and damages the skull. Its narrow field of vision and shallow imaging area limit the observation of subcortical brain areas, so it is impossible to observe the glymphatic system from the whole brain field (Palczewska et al., 2023). Compared with TPI, transcranial optical imaging does not damage animals’ skull in the experimental process. Transcranial optical imaging can distinguish the region 1∼2 mm below the surface of the skull and can detect the fluorophore 5 ∼ 6mm below the surface of the cortex. It can complete the wide-area imaging of CSF flow in the dorsal perivascular of the cerebral cortex of living mice (Li and Wan, 2021). However, it can only observe the dorsal region of the cerebral cortex and cannot image the ventral area of the cerebral cortex (Sweeney et al., 2019). MRI can dynamically monitor the whole brain in real-time and facilitate studying CSF flow in the glymphatic system. The recently discovered NIRF can perform non-invasive measurement of human brain fluid dynamics, which is conducive to long-term brain monitoring (including sleep time) and can be combined with different magnetic neuroimaging technologies (Liu Y. et al., 2022). It can perform real-time dynamic detection of CSF tracer *in vivo*, but its low spatial resolution makes it impossible to analyze the fluid flow in perivascular space (Gakuba et al., 2018). MRI (Iliff et al., 2013a; Stanton et al., 2021), single-photon emission computerized tomography (SPECT), and positron emission tomography (PET) coupled with computerized tomography (Benveniste et al., 2021) can be used to obtain brain-wide 4D images of tracer movement *in vivo* in experimental animals. Notably, these methods are readily translatable to clinical neuroimaging studies. Details on preclinical methods to study the glymphatic system are shown in Table 1.

**TABLE 1:** Details on preclinical methods to study glymphatic cerebrospinal fluid flow.

Study method		Application	Advantages	Disadvantages
***Ex vivo* technique**				
Brain or spinal cord fixed coronal sections		The influx of CSF tracers into brain parenchyma was assessed with several slices	Combined with immunohistochemistry to compare CSF flow with the expression pattern of related proteins.	Fault location of CSF tracers due to the collapse of perivascular space in dead animals; Destruction of tissue by dissection; Time-consuming.
Transparent mice’s panoptic imaging (3DISCO) (Cai et al., 2019)		Imaging the whole rodent head or body	Directly show the connection between the brain and meningeal	Fault location of CSF tracers due to the collapse of perivascular space in dead animals; Time-consuming and limited quantifiability; Immunolabelling is difficult
***In vivo* technique**				
Two-photon Fluorescence Imaging(TPI)		To observe the diffusion of CSF tracer along the outside of cortical surface arteries and penetrating arterioles	High spatial resolution	Invasive to the study object; Narrow field of vision and shallow imaging area limit the observation of subcortical brain areas, and it is impossible to observe the glymphatic lymphatic system from the whole brain field.
Near-infrared fluorescence(NIRF) imaging		Realize real-time dynamic detection of CSF tracer in living animals	Meet the needs of in vitro and in vivo research	With low spatial resolution, fluid flow in perivascular spaces cannot be analyzed
Transcranial optical imaging		Wide-area imaging of CSF flow in dorsal perivascular of the cerebral cortex of living mice	Skull integrity is not destroyed	It can only observe the dorsal of the cerebral cortex and cannot image the ventral area of the cerebral cortex.
PET/SPECT in combination with computed tomography (Benveniste et al., 2021)		The influx of tracers in large CSF spaces, the influx into the brain, and efflux from the brain parenchyma	Quantitative and dynamic imaging of the whole CNS and body	Low spatial resolution; Computed tomography provides anatomical information mainly for hard tissue (for example, bone); Limited possibilities for physiological interventions and monitoring during a dynamic scan
Magnetic resonance imaging(MRI)	Dynamic Contrast-Enhanced MRI(DCE-MRI)	It reflects the inflow and outflow of CSF in the glymphatic system, which is completed by the rate of the contrast agent entering the brain parenchyma and elimination from the brain parenchyma.	3D visualization of the flow of CSF in the whole brain; Provide time and space information at the same time	Spatial resolution does not allow analysis on the micro-scale; The distribution of the tracer in the brain cannot be monitored in real-time; The contrast agent will deposit in the brain parenchyma for a long time, thus causing adverse effects.
	Diffusion Tensor Imaging (DTI)	To study fluid flow in perivascular space	A contrast agent is not required; Non-invasive imaging	Low spatial resolution, can not perform microscopic analysis; Unable to image in real-time
	Chemical exchange saturation transfer-MRI (CEST-MRI)	Reflect the changes of information at the molecular level, such as glucose and protein	It can capture protein and other molecular information and conduct metabolic function measurements.	The specific absorption rate is high, and there are hidden dangers when applied to human body research; Low resolution, unable to conduct microscopic analysis

## 6. Discussion

The concept of the glymphatic system enriches the theory of fluid circulation and waste clearance in the brain. In the past, CSF was thought to be produced from the choroid plexus and circulated in subarachnoid space. However, the glymphatic system provides the hypothesis that CSF enters brain parenchyma and exchanges with ISF dynamically. When the exchange balance is influenced, ISF might be another CSF reserve (Jessen et al., 2015). In the past, researchers thought there was no brain lymphatic system, and the brain’s removal of wastes in the brain was mainly completed through CSF circulation and its addition to blood circulation (Syková and Nicholson, 2008). In fact, the brain parenchyma does not come into direct contact with CSF, and CSF circulation may not be efficient enough due to the limitation of the molecular weight of metabolites. The glymphatic system establishes a bridge between CSF and brain parenchyma, which can help explain brain waste’s clearance pathway.

The discovery of the glymphatic system provides a new direction for understanding brain diseases, which shifts the focus from the changes in the specific structure of the brain to the overall fluid circulation in the brain. A case in point is the critical role of the glymphatic system in understanding the occurrence of brain edema. Traditionally, brain edema is thought to be formed entirely by fluid accumulation in the intravascular compartment (Go, 1984). However, relevant studies of the glymphatic system have found that CSF is the primary source of initial brain edema rather than blood leakage (Mestre et al., 2020). CSF enters the brain parenchyma along the perivascular space within a few minutes after ischemic injury (Mestre et al., 2020). This process is initiated by ischemic spreading depolarization and subsequent vasoconstriction, which expands the perivascular space and doubles the rate of glymphatic inflow. The entry of CSF through the PVS may promote the swelling of astrocytic endfeet and pericyte contraction, which further reduces blood flow and induces the development of an infarction area (Ito et al., 2011; Khennouf et al., 2018). Moreover, the concept of the glymphatic system coincides with the holistic perspective and integrity of the therapeutic effect of traditional Chinese medicine. Traditional Chinese medicine often uses two or more herbs through herbal compatibility to treat diseases, different herb components play different roles in treating brain diseases and finally have a good curative effect when combined. With the knowledge of the glymphatic system, traditional Chinese medicine may better explain multi-ingredient multi-target efficacy. Traditional Chinese medicine formulas may treat diseases by regulating vasoconstriction and relaxation, changing PVS space (Song et al., 2018), and impacting the polar distribution of AQP4 (Yang et al., 2019) and astrocyte swelling.

The proposal of the glymphatic system also provides a new direction for delivering medication. The existence of the BBB and B-CSFB helps to prevent pathogens and toxic substances from entering the brain. However, they also prevent the entry and clearance of medicine for treating brain-related diseases. New ways of medicine delivery have emerged based on the understanding of the glymphatic system. Indocyanine green (ICG) - loaded PLGA nanoparticles were injected near the regional lymph node in the neck of mice. The particles would first converge in the regional deep cervical lymph nodes, then be efficiently transported to the brain through meningeal lymphatic vessels, significantly inhibiting glioma growth in mice and prolonging the survival period of treatment. The uptake of drugs injected with this method in the brain is 44 times higher than that by intravenous injection (Zhao et al., 2020). Drug delivery systems (DDS) are established to conquer the BBB and transport drugs into the brain. However, the elimination route of DDS is unclear. In an experiment, nano-sized DDS in the brain was systematically tracked, and it suggested that it was critically drained by the glymphatic system from the blood vessel basement membrane to periphery circulations (Liu R. et al., 2022). Although their studies are at the beginning stage and drug administration or clearance is affected by many factors (the polarity and safety of drugs are difficult to control), these studies still provide a new direction for medicine delivery.

However, the theory of the glymphatic system is still controversial. Firstly, some researchers believe that the function of the glymphatic system is exaggerated. For instance, several studies have proved that CSF flows along the peripheral spaces of large vessels in convective flow or dispersion and then regulates CSF / ISF exchange in diffusion. This is inconsistent with the fact that CSF / ISF exchange is realized through bulk flow in the glymphatic system (Asgari et al., 2016; Holter et al., 2017; Abbott et al., 2018; Hannocks et al., 2018). Secondly, the role of capillaries in CSF/ISF exchange between arterioles and venules was neglected in glymphatic system theory. Some experiments have proposed that CSF/ISF convection around capillaries occurs in the basal lamina, connects the periarteriolar and the perivenous space, and forms an adequate convective circulation pathway (Hannocks et al., 2018; Pizzo et al., 2018). This structure is more efficient for maintaining neuronal microenvironment homeostasis and promotes substance exchange in CSF / ISF (Wolak and Thorne, 2013). Last but not least, the study on the functional regulation of the glymphatic system is only based on rodent animal models and has not been applied to humans. Thus, we need follow-up experiments to prove that the glymphatic system is also important for the steady-state maintenance of the human brain by supplementing subsequent experiments.

Moreover, there are still some situations that the glymphatic system has not explained. In some diseases like AD or PD, it is not clear whether the malfunction of the glymphatic system occurs first or whether the relevant pathological changes like Aβ or tau-protein deposit happen in the first place. This is crucial to understanding the etiology of diseases. Besides the exchange between CSF/ISF, will the balance be influenced by diseases or altered in some scenarios?

In a word, the proposal of the glymphatic system breaks the traditional understanding that there is no lymphatic system in the brain, complements the theory of fluid circulation and metabolic pathways of brain wastes, and draws a lot of attention to the critical role of fluid circulation in maintaining the homeostasis of the brain environment, which is of great significance. However, there are also many controversies and unsolved problems in the glymphatic system, which requires more researchers to engage in the field for further study, constantly innovate and improve the research paradigm and methods, and make better use of the results of the glymphatic system, to play a more significant role in treating related diseases.

## Author contributions

All authors listed have made a substantial, direct, and intellectual contribution to the work, and approved it for publication.
